# Laser Interstitial Thermal Therapy in Patients with Newly Diagnosed Glioblastoma: A Systematic Review

**DOI:** 10.3390/jcm10020355

**Published:** 2021-01-19

**Authors:** Ilaria Viozzi, Alis Guberinic, Christiaan G. Overduin, Maroeska M. Rovers, Mark ter Laan

**Affiliations:** 1 Department of Neurosurgery, Radboud University Medical Center, Radboud Institute for Health Sciences, 6525 GA Nijmegen, The Netherlands; Ilaria.viozzi1@radboudumc.nl (I.V.); Alis.Guberinic@radboudumc.nl (A.G.); 2 Department of Radiology, Radboud University Medical Center, Radboud Institute for Health Sciences, 6525 GA Nijmegen, The Netherlands; Kristian.Overduin@radboudumc.nl; 3 Departments of Health Evidence and Operating Rooms, Radboud University Medical Center, Radboud Institute for Health Sciences, 6525 GA Nijmegen, The Netherlands; Maroeska.Rovers@radboudumc.nl

**Keywords:** LITT, laser ablation, glioblastoma

## Abstract

Background: Laser interstitial thermal therapy (LITT) is a minimal invasive neurosurgical technique for the treatment of brain tumors. Results of LITT have been reported in a case series of patients with deep seated and/or recurrent glioblastoma or cerebral metastases. With this review we aim to summarize the currently available evidence regarding safety and effectiveness of LITT in patients with newly diagnosed glioblastoma (nGBM). Methods: A literature search was performed using electronic databases (PubMed and Embase). Papers were assessed for the methodological quality using the Risk Of Bias In Non- randomised Studies - of Interventions (ROBINS-I) tool, and the Grading of Recommendations Assessment, Development and Evaluation (GRADE) was used to assess the quality of the evidence. Results: We identified 835 papers of which only 11 articles were eligible for our review. All papers suffered from serious or critical risk of bias, and the quality of evidence was graded as very low according to the GRADE criteria. None of the studies was randomized and reporting of confounders and other parameters was poor. Median overall survival (OS) ranged from 4.1 to 32 months and progression free survival (PFS) from 2 to 31 months. The mean complication rate was 33.7%. No quality of life or cost-effectiveness data were reported. Conclusions: Due to the low quality of the studies, it is not possible to draw firm conclusions regarding the (cost) effectiveness of LITT in patients with newly diagnosed glioblastoma. The low quality of evidence shows the need for a well-designed prospective multicenter randomized controlled trial.

## 1. Introduction

Laser interstitial thermal therapy (LITT) has emerged as an alternative, minimal invasive neurosurgical technique for patients with epilepsy, tumors or radiation necrosis [1]. LITT has been proven to be useful for lesions located in difficult to access brain areas, in patients with a greater surgical risk or in the case of recurrence and repeated resections [1,2,3,4,5,6,7].

Patients with a newly diagnosed glioblastoma (nGBM) who are not amenable for surgery and only receive chemoradiation miss the benefit of surgical resection. Survival is significantly worse for these patients as compared to those who undergo subtotal or gross total resection [8]. Patients with unresectable glioblastoma could especially benefit from LITT, as LITT can be added to the standard adjuvant chemotherapy and radiotherapy to improve clinical outcomes. Previous studies have suggested that patients with nGBM have reasonable survival outcomes and complication rates when treated with LITT [1,2,3,4,5,7,9,10]. In addition, LITT is not bound to a maximum dose and can therefore be used multiple times [11]. Furthermore, patients treated with LITT seem to need a shorter length of hospital stay and recover faster than patients undergoing an open craniotomy [12]. It has also been advocated that LITT could temporarily increase permeability for chemotherapeutic agents by disrupting the blood–brain barrier [13].

So far, several case-series studies have been published and LITT is already at use, especially in the US, but nevertheless its effectiveness in patients with nGBM remains unclear. Two recent systematic reviews reported good results for patients with recurrent glioblastoma [14] and recurrent brain metastases [15] treated with LITT. A previous systematic review published in 2016 on nGBM treated with LITT included only three studies [5].

With this systematic review we aim to summarize current available data on the safety and effectiveness of LITT in adult patients suffering from nGBM.

## 2. Material and Methods

### 2.1. Search of the Literature

PRISMA guidelines (preferred reporting items for systematic reviews and meta-analyses) were followed to conduct and report this systematic literature review [16].

We performed a broad systematic literature search in Medline and Embase for all studies investigating the application of LITT in newly diagnosed glioblastoma (nGBM). We searched for studies published up to 17 December 2020, combining the following terms: “Laser interstitial thermal therapy” and synonyms AND “Glioblastoma” and synonyms. The search phrase was kept broad to capture all potentially relevant articles (see Appendix A). In addition, bibliographies of retrieved papers were searched. Duplicate articles were eliminated using Endnote X9 bibliographic database (Clarivate Analytics, Boston, MA, USA).

### 2.2. Study Selection

We included studies investigating LITT in supratentorial nGBM in adult patients (>18 years old) reporting on effectiveness and/or safety and/or cost-effectiveness. No limits were set for languages. Exclusion criteria were the following: (1) review articles, (2) single case reports, (3) conference abstracts, (4) animal studies or in-vitro studies, (5) case series reporting only other intracranial lesions or only recurrent GBM treated with LITT, (6) case series where it was not feasible to extract data of nGBM patients only and (7) case series on the same dataset.

Some of the screened studies were conducted at the same institutions and partially overlapped in inclusion criteria. We contacted the authors of these studies for clarifications [2,4,17,18]. If two or more articles showed patients overlap, we included the article with the most complete outcomes or with the largest population.

Some studies included both patients with nGBM and patients with other pathologies (recurrent glioblastoma, astrocytoma WHO III, metastases and epilepsy) and reported aggregated results for all patients. These studies were included in this systematic review if it was possible to extract OS, PFS or complications specifically for nGBM patients.

Titles and abstracts were scanned, and studies were included based on the full text by two independent reviewers (IV and AG); disagreements were resolved with a third reviewer (MtL). Rayyan web-based software was used to assist in the process of screening and selection of abstracts and articles.

### 2.3. Data Extraction

Two authors (IV and AG) extracted data on study characteristics (publication year, years of inclusion and study design), patient characteristics (age, sex, KPS before and after LITT), tumor characteristics (location, histological diagnosis, IDH mutation, MGMT methylation and tumor volume before and after LITT), overall survival (OS), progression free survival (PFS), quality of life (QoL), complications, mortality and hospital stay, if reported.

### 2.4. Quality Assessment

The risk of bias was evaluated by both reviewers (IV and AG) independently using the validated The Risk of Bias in Non-randomized Studies—of Interventions (ROBINS-I) assessment tool (version for cohort-type studies). The ROBINS-I outlines seven domains of biases divided in the preintervention phase (bias due to confounding and bias due to selection of participants), at the intervention phase (bias in classification of interventions) and post-intervention phase (deviation from intervention, missing data, bias in measurement of outcomes and selection of reported results). Any disagreements were resolved by discussion with a third reviewer (MtL).

The GRADE methodology was used to assess the quality of the body of retrieved evidence (GRADEpro, Version 20. McMaster University, 2014).

### 2.5. Data Analysis

We planned to pool the primary outcome data on survival outcomes and complications from all the studies. However, due to large heterogeneity between studies and inconsistent reporting of confounders, pooling was not possible. We extracted median OS and median PFS where reported. A Kaplan Meier analysis was conducted to calculate median OS and PFS based on individual patients with newly diagnosed glioblastoma data where reported in the articles. Ranges of median OS and PFS survival and mean complication rate and perioperative mortality rate per study are reported.

## 3. Results

**Figure 1:**
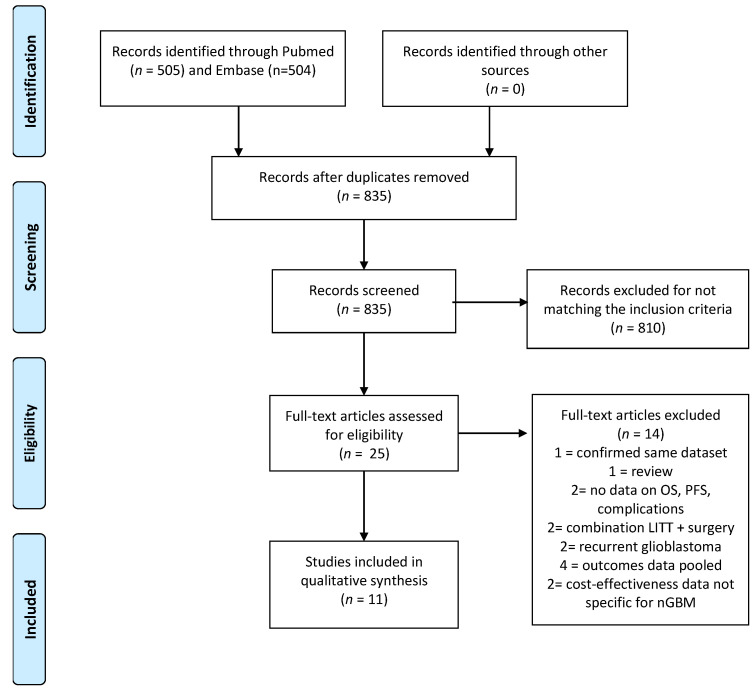
Preferred reporting items for systematic reviews and meta-analyses (PRISMA) flow-chart. OS: overall survival; PFS: progression free survival; LITT: laser interstitial thermal therapy; nGBM: newly diagnosed glioblastoma.

Our broad systematic search regarding the use of LITT in patients with glioblastoma yielded 835 articles after removing duplicates (Figure 1). Based on the title and abstract, 810 articles were excluded due to incompatibility with our eligibility criteria (review studies; conference abstracts; studies regarding LITT in patients with a diagnosis other than nGBM (recurrent glioblastoma, metastases, radiation necrosis or other intracerebral tumors and studies regarding LITT in patients with epilepsy) and studies regarding other forms of laser ablation). Full texts of the remaining 25 studies were screened and another 14 articles were excluded (4 articles reported combined results for recurrent and primary glioblastoma and/or glioblastoma with anaplastic astrocytoma, 2 articles did not report on survival or complications, 1 article reported preliminary results of another included study, 2 studies reported only data about recurrent glioblastoma, 2 studies investigated the combination of LITT with surgical resection, 1 study was a systematic review on the cost-effectiveness and 2 articles reporting on cost-effectiveness not specific for nGBM). A total of 11 articles reporting data on clinical outcomes were included in this systematic literature review [1,3,19,20,21,22,23,24,25,26,27].

### 3.1. Risk of Bias Assessment

Nine out of the 11 studies scored a serious risk of bias in the overall methodological quality assessment, while the remaining two scored a critical risk of bias. The main reason for the high risk of bias is related to the study design (all studies except for one [19] are retrospective studies, leading to a serious risk of bias in selection of participants) and to lack of reporting of confounders, leading to a serious or critical risk of bias. The individual biases for each article ranged from critical to low and are depicted in Table 1.

### 3.2. GRADE Assessment

None of the included articles randomized the participants. Additional risks for biases were retrospective inclusions and conflicts of interest. Only half of the articles reported a 95% confidence interval for the point estimates. It was therefore not possible to measure whether the intervals overlapped. The small sample sizes and broad 95% CI made the evidence also prone for imprecision. Considering these limitations, the quality of evidence for all included studies was downgraded to very low (Table 2).

### 3.3. Clinical Outcomes

All included studies were conducted in the US, with a great majority using the Neuroblate–Monteris system (81%). None of the included studies was randomized and only one reported data from a retrospective historical cohort as the control group [3]. Except for one [19], all of the studies were retrospective, and except for two [3,24] all were single center studies. Only two studies investigated specifically nGBM [3,27], while the rest presented also data from other pathologies. Study characteristics and baseline patients’ characteristics are reported in Table 3.

The eleven included studies included a total of 114 patients with newly diagnosed glioblastoma (nGBM) who underwent LITT as upfront treatment. A mean age of 56.5 years (range 34–78) and a predominance of male patients (56.5%) were reported. Most of the treated tumors (61.8%) were deep-seated (thalamus, basal ganglia, corpus callosum and insula). Laterality was reported in five studies for 28 patients (10 right side, 13 left side and 5 bilateral). The range of included patients varies from 2 to 24.

Table 4 summarizes survival outcomes and shows reported confounders. Median OS was reported or calculable in all studies, while progression free survival was reported in 9 of 11 studies. The complication rate was reported in nine studies. Reporting of possible confounders was much less consistent. KPS was reported or could be extracted in 7 studies, IDH mutational status in 5, MGMT hypermethylation in 3, age in 9, tumor location in 10, tumor volume in 10, hospital stay in 5 and adjuvant treatment in 7 studies.

The range of median overall survival varied between 3.3 and 32.3 months. The range of median progression free survival varied between 2 and 31.9. Further analysis was not conducted because of the broad ranges, the inconsistency in reporting confounders and the heterogeneity in included patients. Mean preoperative KPS reported for 66 patients was 80. Adjuvant treatment after LITT was reported for 81 patients: most of them underwent adjuvant treatment (88%). The chosen adjuvant treatment was reported very inconsistently. Most of the studies report using standard chemotherapy with temozolomide and radiotherapy.

Table 5 shows reported complications, mortality and hospital stay. In the 9 studies reporting complications in nGBM patients, 25 complications were described in 74 patients (33.7%). The most common complication was increased new neurological deficits (64%). Three cases of perioperative mortality were described in 74 patients treated for nGBM (4%): 2 cases due to intracerebral hemorrhage and 1 case to meningitis. Mean tumor volume reported for 74 patients was 14.8 cc (range 1.3–62.77 cc). There were no data on post-ablation tumor volume. Mean hospital stays reported for 50 patients were 4.6 days.

There were no data on preoperative and/or post-operative quality of life.

### 3.4. Cost-Effectiveness

This systematic review yielded no articles reporting data on cost-effectiveness of LITT in patients with nGBM.

## 4. Discussion

This review shows that high quality evidence regarding the safety and clinical effects of LITT as upfront treatment for patients with nGBM is lacking.

The reported median OS and PFS of all studies varied significantly in range. Since confounders were underreported, it was not possible to correlate survival with preoperative KPS, IDH mutation, MGMT methylation and therefore further analysis of these data was not possible. We could only conclude that the reported range varied between 3.3 and 32.3 months for median overall survival and between 2 and 31.9 for PFS. The broad range in survival outcomes was in line with results reported from other systematic review of LITT in recurrent glioblastoma and recurrent brain metastases [14,15]. Ivan et al. reported in a systematic review and meta-analysis from 2016 an overall survival of 14.2 months (range 0.1–23 months), but they included only three studies and both patients with glioblastoma and astrocytoma WHO grade III, who mostly show a better prognosis than patients with glioblastoma [5]. It is remarkable that many studies report survival based on pooled results from different pathologies, which raises the question whether these data are reliable. One study from Mohammadi et al. showed comparable survival outcomes in patients treated with LITT as upfront therapy for nGBM and a matched cohort of biopsy-only patients (14.4 months vs. 15.8 months), but given the small population, the study might be underpowered [3]. As no randomized controlled trial has been performed yet, the real effect of LITT on survival is unclear.

As evidence to perform LITT instead of surgical resection in patients with newly diagnosed glioblastoma is lacking, LITT is for now used to treat patients where a resection is not deemed feasible. To compare these results to the current standard of care of patients with unresectable glioblastoma, we searched for literature investigating survival in glioblastoma patients undergoing a biopsy only, followed by adjuvant chemotherapy and radiotherapy as treatment. Current literature shows that nGBM patients undergoing biopsy only face a poorer prognosis than patients undergoing surgical resection. A large meta-analysis from Brown et al. showed significant improvement in overall survival for patients undergoing resection compared with biopsy alone (1-year survival RR, 0.77; 95% CI, 0.71–0.84; and 2-year survival RR, 0.94; 95% CI, 0.89–1.00). In the same meta-analysis, resection appeared to reduce the risk for progression compared with biopsy alone at 6 months (RR, 0.61; 95% CI, 0.44–0.84) [8]. Kole et al. published results from a large cohort including more than 1300 patients with nGBM undergoing biopsy followed by adjuvant chemoradiotherapy and reported a mean OS of 9.2 months (8.6–9.9) [28]. Another meta-analysis investigated overall survival in elderly patients with high grade glioma and revealed an overall survival of 5.71 months (95% CI 5.04–6.36) for patients treated with biopsy [29]. Data from the Dutch Quality Registry Neurosurgery revealed a mean OS of 5.1 months (95% CI 137–176 days) for patients undergoing biopsy only for nGBM in our country [30], indicating that also reported OS rates for standard of care show significant variation.

LITT could offer selected patients with unresectable glioblastoma a treatment option, providing cytoreduction where surgery is impossible. Potential advantages of LITT comprise its minimal invasiveness, the possibility of treating deep lesions, the ability to repeat treatment and possibly chemotherapy could be started sooner after LITT then after surgery. The majority of patients included in our review could receive adjuvant chemotherapy and radiotherapy after LITT, which indicates they preserved a good KPS and that LITT did not interfere with the post-operative standard treatment. The role of LITT in transiently disrupting the permeability of the BBB and thus theoretically improving the response on chemotherapy has been suggested but not demonstrated yet [13].

The mean complication rate of LITT in nGBM from all articles was 33.7%. In previously published studies, where results for LITT were pooled for different patient populations (newly and recurrent glioblastoma, metastases and epilepsy), complication rates vary between 13 and 26% [4,5,12]. A possible explanation for this result is the focus of our systematic review on nGBM only. Possibly the included studies reported more than average deep seated tumors (61.8% in thalamus, basal ganglia, corpus callosum and insula) explaining more neurological complications, but since data on tumor location, volume and complications were inconsistently reported, further analysis was not possible and we could only speculate. It is reported that these complications concern transient neurological deficits. Three cases of mortality were reported among 71 patients, in two cases due to intracranial hemorrhage and in one case due to meningitis.

The mean lesion size from all reported studies was 14.8 cm^3^, which is higher than elsewhere reported to be safe targets for ablation [7,31]. In our systematic review it is not possible to correlate complication rate to tumor volume or other confounders (age, Karnofsky performance score—KPS and comorbidities) given the lack of data.

To our knowledge, this is the largest and most comprehensive systematic review on LITT in nGBM. Unfortunately, we were unable to perform a quantitative analysis or a best-evidence synthesis, due to the heterogeneity and low quality of the evidence.

Another limitation is the relative low number of included articles despite the extensive literature search. It is remarkable that many studies overlapped in inclusion criteria and inclusion period. We tried to exclude articles with the same populations, but still some overlapping is possible.

The addition of LITT to the standard treatment for patients with an nGBM could be promising in cases when surgery is deemed unsafe or undesirable. Rates of major complications seem acceptable, but given the lack of high-quality trials, current data are insufficient to demonstrate (cost-)effectiveness of LITT for nGBM, and in our opinion should therefore not be offered to patients outside clinical trials.

In line with these findings, we will perform a randomized pilot study to compare LITT to biopsy only in patients with unresectable glioblastoma, followed by adjuvant chemoradiotherapy according to the standard guidelines (Radboudumc, Nijmegen, The Netherland (Clinicaltrials.gov ID: NCT04596930)). If data on safety and feasibility will be satisfying, we aim to perform a multicenter randomized controlled trial in The Netherlands to investigate cost-effectiveness of LITT.

## 5. Conclusions

Our systematic review confirms the lack of high-quality comparative studies to provide robust data on (cost-)effectiveness of LITT in patients with newly diagnosed glioblastoma. The reported studies are of very low quality and although LITT seems safe, it is too early to recommend LITT treatment for nGBM patients. In line with previous reviews, this shows the need for a well-designed prospective multicenter randomized controlled trial.

## Figures and Tables

**Table 1:** Risk of bias according to the ROBINS-I tool.

Author	Confounding	Selection of Participants	Classification of Intervention	Deviation from Intervention	Missing Data	Measurement of Outcomes	Selection of Reported Results	Overall
**Hawasli et al. #**	S	L	L	NR	L	L	NR	Serious
Schroeder et al.*	S	S	L	NR	L	L	NR	Serious
**Thomas et al.**	S	S	L	NR	L	L	NR	Serious
**Rennert et al.**	C	S	L	NR	L	L	NR	Critical
**Kamath et al. #**	C	S	L	NR	L	L	NR	Critical
Beaumont et al. *	S	S	L	NR	L	L	NR	Serious
Mohammadi et al. *	M	S	L	NR	L	L	NR	Serious
**Shah et al.**	S	S	L	NR	L	L	NR	Serious
**Kamath et al. #**	S	S	L	NR	L	L	NR	Serious
Murayi et al. *	S	S	L	NR	L	L	NR	Serious
Jamshidi et al. *	M	S	L	NR	L	L	NR	Serious

S: serious risk of bias (orange); C: critical risk of bias (red); M: moderate risk of bias (yellow); L: low risk of bias (green). NR = Not reported: data missing given the lack of control group (gray). #: might include part of the same population. *: might include part of the same population. NR: not reported.

**Table 2:** GRADE assessment of quality of evidence.

The Efficacy and Safety of Laser Interstitial Thermal Therapy on Patients Suffering from Supratentorial Newly Diagnosed Glioblastoma			
**Patient or population: patient older than 18 years suffering from supratentorial newly diagnosed glioblastoma.** **Intervention: stereotactic laser ablation.**			
**Primary outcomes**	**The point estimate**	**No. of participants (studies)**	**Quality of the evidence (GRADE)**
**Overall survival**	The point estimate is 10.2 months. The medians ranged from 3.3 to 32 months.	111 (10 non-RCT)	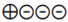 Very low ^a,b,c^
**Progression free survival**	The point estimate is 7 months. The medians ranged from 2 to 31.9 months.	106 (9 non-RCT)	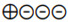 Very low ^a,b,c^
**Complications**	The point estimate is 33.7%.	111 (11 non-RCT)	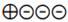 Very low ^a,b,c^
GRADE Working Group grades of evidence (GradePro). High quality: we are very confident that the true effect lies close to that of the estimate of the effect. Moderate quality: we are moderately confident in the effect estimate: the true effect is likely to be close to the estimate of the effect, but there is a possibility that it is substantially different. Low quality: our confidence in the effect estimate is limited: the true effect may be substantially different from the estimate of the effect. Very low quality: we have very little confidence in the effect estimate: the true effect is likely to be substantially different from the estimate of effect.			

^a^ Risk of bias: no blinding of therapists and participants (not possible), and no randomization. ^b^ Imprecision: small number of participants and broad CI. ^c^ Selection bias: retrospective patient selection.

**Table 3:** Study characteristics and patients’ baseline. First author, study design, year of publication are reported; number of cases of nGBM, sex, mean age with range or CI, tumor location and technology used are reported.

Author	Study Design	Year	nGBM	Sex M/F	Mean Age (Range)	Tumor Location (No)	Laterality	Surgical Technology
**Hawasli et al. #**	prospective single center case-series	2013	6	4/2	46.7 (34–78)	thalamus (4), basal ganglia (1), corpus callosum (1)	NR	Neuroblate
Schroeder et al.*	retrospective single center case-series	2014	3	1/2	54.6 (34–72)	basal ganglia and thalamus (not further specified)	Right (2) Left (1)	Neuroblate
**Thomas et al.**	retrospective single center case-series	2016	8	NR	60.8	corpus callosum (5), insula (2), thalamus (1)	NR	Neuroblate/Visualase
**Rennert et al.**	retrospective single center case-series	2016	2	0/2	58. 5 (CI 52–65)	parietal (2)	Left (2)	Neuroblate
**Kamath et al. #**	retrospective single center case-series	2017	23	NR^	NR^	deep-seated (9) lobar (14)	NR	Neuroblate
Beaumont et al.*	retrospective multicenter case-series	2018	9	7/2	56.4 (40–69)	corpus callosum (9)	Right (5) Left (2) Bilateral (2)	Neuroblate
Mohammadi et al.*	retrospective multicenter case-series compared with historical cohort	2018	24	12/12	54	lobar (11), deep (13)	NR	Neuroblate
**Shah et al.**	retrospective single center case-series	2019	11	7/4	59 (40–71)	frontal (4), frontoparietal (2), Parietal (2), temporal (2), thalamus (1)	Right (3) Left (8)	Visualase
**Kamath et al. #**	retrospective single center case-series	2019	17	NR^	NR^	NR^	NR	Neuroblate
Murayi et al.*	retrospective single center case-series	2020	8	5/3	55.25 (34–72)	thalamus (8)	NR	Neuroblate
**Jamshidi et al.**	retrospective single center case-series	2020	3	2/1	64 (59–73)	corpus callosum (3)	Bilateral (3)	Visualase

#: might include part of the same population. *: might include part of the same population. NR: not reported. NR^ data pooled from different populations. CI: confidence interval. Mean age is reported in years.

**Table 4:** Overall survival (OS) and progression free survival (PFS) are reported with respective range of CI 95% when reported in the original studies.

Author	KPS Pre-LITT	IDH Mut	MGMTmet	Median OS (Months) (Range or CI 95%)	Median PFS (Months) (Range or CI 95%)	Adjuvant Treatment (Yes)
**Hawasli et al. #**	NR^	NR	NR	4.1 (0.1–10.7)	2.9 (2.6–3.2)	4/6
Schroeder et al.*	80 (70–90)	NR	NR	8.8 (2.6–15)	NR	NR
**Thomas et al.**	85	0%	NR	8 (0–15)	2	7/8
**Rennert et al.**	NR	50%	NR	6.1 (6–21.8)	NR	NR
**Kamath et al. #**	NR	NR	NR	11.4	5.9	NR
Beaumont et al.*	80 (60–90)	0	42%	7 (0.4–23.8)	3.1 (0.4–9.1)	12/15
Mohammadi et al.*	80 (60–90)	0	30%	14.4	4.3 (CI 3.3–5.7)	24/24
**Shah et al.**	80	NR	NR	32.3	31.9	NR
**Kamath et al. #**	NR	NR^	NR^	9.1 (CI 4.2–14.2)	3.6 (CI 0.37–7.67)	15/17
Murayi et al.*	80 (60–90)	NR	NR	3.3 (0.9–34)	2.7 (0.9–11.7)	7/8
**Jamshidi et al.**	80 (80–90)	0%	33%	8	mean 3.55	2/3

#: might include part of the same population. *: might include part of the same population. NR: not reported. NR^ data pooled from different populations. KPS: Karnofsky Performance Scale. IDH mut: Isocitrate dehydrogenase mutation. MGMTmet: methylguanine methyltransferase. OS: overall survival. PFS: Progression free survival. CI: confidence interval.

**Table 5:** Complications, tumor volumes, mortality and hospital stay.

Author	Complications	Tumor Volumes (Range)	Type of Complication	Mortality (Reason)	Hospital Stay (Days) (Mean, Range)
**Hawasli et al. #**	3/6	17.7 (3.2–42.2)	Neurological deficits (2), meningitis (1)	1/6 (meningitis)	9 (3–29)
Schroeder et al.*	2/3	14.3 (5.8–27.8)	Neurological deficits (1), ICH (1), DVT/PE (1)	1/3 (ICH)	NR
**Thomas et al.**	1/8	22.4	Functional decline (1)	0/8	NR
**Rennert et al.**	0/2	10.1 (7–13.2)	None	0/2	NR
**Kamath et al. #**	NR^	NR	NR^	NR^	NR^
Beaumont et al.*	6/9	25.4 (8.28–62.77)	Neurological deficits (4), hydrocephalus (1), edema (1)	0/9	3 (1–11)
Mohammadi et al.*	9/24	9.33 (1.31–62.78)	Neurological deficits (6), ICH (2), DVT (1)	0/24	3 (1–26)
**Shah et al.**	0/11	6.8 (1.2–127.0)	None	0/11	NR^
**Kamath et al. #**	NR^	NR	NR^	NR^	NR^
Murayi et al.*	4/8	12.5 (2–22.1)	Neurological deficits (3), hydrocephalus (1)	1/8 (ICH)	5.3 (1–19)
**Jamshidi et al.**	0/3	14.95 (6.32–23.48)	None	0/3	3 (2–5)

#: might include part of the same population. *: might include part of the same population. NR: not reported. NR^: data pooled from different populations. ICH: intracranial hemorrhage. DVT: deep vein thrombosis. PE: pulmonary embolism.

## Appendix A

Search strategy PubMed: (((“Laser Therapy”[Mesh:NoExp] OR LITT[Title/Abstract] OR stereotactic laser[tiab] OR stereotaxic laser[tiab] OR Laser ITT[tiab] OR interstitial thermal therapy[tiab] OR laser-induced thermal therapy[tiab] OR interstitial thermotherapy[tiab] OR laser thermotherapy[tiab])) AND ((“Brain Neoplasms”[Mesh] OR “Glioblastoma”[Mesh] OR glioma*[tiab] OR glioblastoma*[tiab] OR Astrocytoma*[tiab] OR ((brain[ti] OR cerebral*[ti] OR intercrani*[ti]) AND (tumor*[ti] OR tumour*[ti] OR neoplas*[ti] OR cancer*[ti] OR malignan*[ti]))))).

Search strategy Embase: Glioblastoma/ or (glioma* or glioblastoma* or astrocytoma* or ((tumor* or tumour* or cancer* or neoplasm* or neoplasia) and (brain* or cerebr* or intercran*))).ti,ab,kw. AND laser surgery/ or laser thermotherapy/ or (LITT or (stereotactic adj3 laser*) or (stereotaxic adj3 laser*) or Laser ITT or interstitial thermal therapy or laser-induced thermal therapy or interstitial thermotherapy or laser thermotherapy).ti,ab,kw.
